# Voltage-gated calcium channel subunit α_2_δ-1 in spinal dorsal horn neurons contributes to aberrant excitatory synaptic transmission and mechanical hypersensitivity after peripheral nerve injury

**DOI:** 10.3389/fnmol.2023.1099925

**Published:** 2023-03-23

**Authors:** Keisuke Koga, Kenta Kobayashi, Makoto Tsuda, Kazufumi Kubota, Yutaka Kitano, Hidemasa Furue

**Affiliations:** ^1^Department of Neurophysiology, Hyogo Medical University, Nishinomiya, Japan; ^2^Section of Viral Vector Development, National Institute for Physiological Sciences, Okazaki, Japan; ^3^Department of Molecular and System Pharmacology, Graduate School of Pharmaceutical Sciences, Kyushu University, Fukuoka, Japan; ^4^Specialty Medicine Research Laboratories I, Daiichi Sankyo Co., Ltd., Tokyo, Japan

**Keywords:** spinal cord, neuropathic pain, α_2_δ-1, synaptic transmission, gabapentinoid, disinhibition

## Abstract

Neuropathic pain, an intractable pain symptom that occurs after nerve damage, is caused by the aberrant excitability of spinal dorsal horn (SDH) neurons. Gabapentinoids, the most commonly used drugs for neuropathic pain, inhibit spinal calcium-mediated neurotransmitter release by binding to α_2_δ-1, a subunit of voltage-gated calcium channels, and alleviate neuropathic pain. However, the exact contribution of α_2_δ-1 expressed in SDH neurons to the altered synaptic transmission and mechanical hypersensitivity following nerve injury is not fully understood. In this study, we investigated which types of SDH neurons express α_2_δ-1 and how α_2_δ-1 in SDH neurons contributes to the mechanical hypersensitivity and altered spinal synaptic transmission after nerve injury. Using *in situ* hybridization technique, we found that *Cacna2d1,* mRNA coding α_2_δ-1, was mainly colocalized with *Slc17a6*, an excitatory neuronal marker, but not with *Slc32a1*, an inhibitory neuronal marker in the SDH. To investigate the role of α_2_δ-1 in SDH neurons, we used clustered regularly interspaced short palindromic repeats (CRISPR)-Cas9 system and showed that SDH neuron-specific ablation of *Cacna2d1* alleviated mechanical hypersensitivity following nerve injury. We further found that excitatory post-synaptic responses evoked by electrical stimulation applied to the SDH were significantly enhanced after nerve injury, and that these enhanced responses were significantly decreased by application of mirogabalin, a potent α_2_δ-1 inhibitor, and by SDH neuron-specific ablation of *Cacna2d1*. These results suggest that α_2_δ-1 expressed in SDH excitatory neurons facilitates spinal nociceptive synaptic transmission and contributes to the development of mechanical hypersensitivity after nerve injury.

## Introduction

Neuropathic pain is an intractable pain condition that appears after nerve damage. Recent studies have revealed the molecular and circuit mechanisms of this abnormal pain symptom (Luo et al., 2014; Peirs and Seal, 2016; Alles and Smith, 2018; Ma, 2022). Gabapentinoids, including pregabalin, gabapentin, and mirogabalin, are among the most commonly recommended drugs for neuropathic pain. Gabapentinoids are known to exert the analgesic effect through binding with α_2_δ-1, a subunit of voltage-gated calcium channels (VGCCs), in the SDH (Field et al., 2006; Taylor, 2009; Kitano et al., 2019). α_2_δ-1–3 subunits are expressed in both the central nervous system (CNS) and peripheral nervous system (PNS; Cole et al., 2005), and gabapentinoids bind to α_2_δ-1 and α_2_δ-2 subtypes; however, binding to α_2_δ-1 is important for its pharmacological effects (Dooley et al., 2007). Previous studies showed that α_2_δ-1 modulates current density and membrane trafficking of VGCCs (Li et al., 2006; Fuller-Bicer et al., 2009; Nieto-Rostro et al., 2018), and α_2_δ-1 in dorsal root ganglion (DRG) neurons is upregulated after nerve injury and enhances axonal transport of VGCCs to their central terminals in the SDH (Dooley et al., 2007; Nieto-Rostro et al., 2018). It was also reported that intrathecal delivery of genetic or pharmacological regents targeting α_2_δ-1 ameliorates mechanical hypersensitivity and aberrant excitatory neurotransmission after nerve injury (Li et al., 2004; Matsuzawa et al., 2014; Alles et al., 2017; Chen et al., 2018), suggesting α_2_δ-1 in the SDH contributes to this pain phenotype. However, as these manipulations affected both SDH and DRG neurons, how α_2_δ-1 expressed in SDH neurons contributes to neuropathic pain phenotype after nerve injury is not determined.

The SDH contains both excitatory and inhibitory interneurons. Although a lot of mechanisms are involved in pain facilitation after nerve injury (Balasubramanyan et al., 2006; Peirs and Seal, 2016; Inoue and Tsuda, 2018), attenuation of inhibitory circuits is considered as one of the prominent mechanisms of pain hypersensitivity after nerve injury (Moore et al., 2002; Coull et al., 2003; Duan et al., 2014; Tashima et al., 2021) and spinal cord injury (Lu et al., 2008), and causes aberrant excitatory synaptic transmission in the SDH (Baba et al., 2003; Todd, 2010; Peirs et al., 2020). However, which types of SDH interneurons express α_2_δ-1 and whether α_2_δ-1 in SDH neurons contributes to the disinhibition-induced enhancement of excitatory synaptic transmission in the SDH is still unknown.

This study aimed to investigate which types of SDH neurons express α_2_δ-1 subunit, and how α_2_δ-1 in SDH neurons contributes to the mechanical hypersensitivity and altered spinal synaptic transmission after peripheral nerve injury. We found that α_2_δ-1 is selectively expressed in excitatory SDH neurons. Furthermore, adeno-associated virus (AAV)-CRISPR-Cas9-mediated α_2_δ-1 knockdown in SDH neurons ameliorated mechanical hypersensitivity and aberrant excitatory synaptic transmission in the SDH after peripheral nerve injury (PNI). Our findings identified the contribution of α_2_δ-1 in SDH neurons to neuropathic pain phenotype after nerve injury and could be a clue to explain why selectivity of α_2_δ-1 is important for pharmacological effect of gabapentinoids.

## Materials and methods

### Animals

Male C57BL/6 J mice (CLEA Japan) and *Vgat-Cre* mice (B6J-*Slc32a1^tm2(cre)lowl^*/MwarJ, Stock No: 028862, The Jackson Laboratory; Vong et al., 2011) were used for all the experiments. All mice were 8–12 weeks old at the start of each experiment and were housed at 22 ± 1°C with a 12-h light–dark cycle with food and water *ad libitum*. All animal studies were reviewed and approved by the Animal Care and Use Committee of Hyogo Medical University. All animal experiments were performed in accordance with the institutional guidelines and were consistent with the ethical guidelines of the International Association for the Study of Pain.

### Adeno-associated virus production and purification

pZac2.1-ESYN-SaCas9 was constructed from the cis-cloning plasmid pZac2.1 with ESYN promoter and pENTER-SaCas9 (Tabebordbar et al., 2016; Koga et al., 2020). Synthetic oligonucleotides included the targeting sequence for *Cacna2d1* Exon 10 (5′-ATCTCGGAGACAGATGTTCGG-3′) or oligonucleotides without target sequence (control) were substituted with the targeting site in the original pENTER-U6-sgBsa1 plasmid (Ran et al., 2015). The resulting U6-sgRNA cassettes (U6-sgCacna2d1 or U6-sgControl) were transferred into pZac2.1-ESYN-mCherry plasmid. AAV9-ESYN-SaCas9, AAV9-ESYN-mCherry-U6-sgCacna2d1 and AAV9-ESYN-mCherry-U6-sgControl were produced using the AAV Helper-Free System (Agilent Technologies) as reported previously (Sano et al., 2020). AAV9-EF1α-FLEX-mCherry (Kohro et al., 2020) was kindly gifted from Prof. Tsuda laboratory. The used viral titers were as follows: AAV9-ESYN-SaCas9, 2.0 × 10^12^ genome copies (GC)/ml; AAV9-ESYN-mCherry-U6-sgCacna2d1 and AAV9-ESYN-mCherry-U6-sgControl, 0.5 × 10^12^ GC/ml; AAV9-EF1α-FLEX-mCherry 1.0 × 10^12^ GC/ml.

### Microinjections

We used previously reported method with some modifications (Kohro et al., 2015). Mice were deeply anesthetized with medetomidine hydrochloride (0.3 mg/kg, Domitol, Meiji Seika Pharma), midazolam (4 mg/kg, Dormicum, Astellas Pharma) and butorphanol (5 mg/kg, Vetorphale, Meiji Seika Pharma). The skin was incised at Th11–L4 and the vertebral column was clamped. The paraspinal muscles around the right side or both sides of the interspaces of Th13-L1 were removed, and the dura mater and the arachnoid membrane were carefully incised to make a small window. rAAV solutions (approximately 500–600 nl in one site) were unilaterally injected into the SDH through the window (at 500 μm lateral from the midline, 200–300 μm depth from the surface). Only for hot-plate test, rAAV solutions were bilaterally injected. We used virus-injected mice 6 weeks or more after microinjection of AAV vectors in the Cas9-mediated knockdown experiments. In the inhibitory neuron labeling experiments, we used virus-injected *Vgat-Cre* mice 3–4 weeks after microinjection.

### Immunohistochemistry

Immunohistochemical (IHC) experiments were performed according to the methods described in our previous study with some modifications (Kohro et al., 2020). Mice were deeply anesthetized by i.p. injection of urethane (1.2–1.5 mg/kg) and perfused transcardially with phosphate buffered saline (PBS), followed by ice-cold 4% paraformaldehyde (PFA)/PBS. The L3-L4 segments of spinal cord, the L4 DRG and/or brain were removed, postfixed in the same fixative overnight at 4°C, and subsequently left immersed in 30% sucrose solution for 2–3 nights at 4°C. Transverse spinal and brain and DRG sections (40 μm thick for brain and spinal cord and 25 μm thick for DRG) were prepared using a cryostat (CM3050S, Leica) and immunostained. Primary and secondary antibodies used are listed below. Primary antibodies: polyclonal mouse anti-NeuN (1:1000, MAB377, Millipore), monoclonal rabbit anti-HA-tag (1:1000, 3,724, Cell Signaling), monoclonal mouse anti-dihydropyridine receptor (α_2_δ-1 Subunit) antibody (1:500 for spinal cord and 1:200 for DRG, D219, Sigma; Taylor and Garrido, 2008; Park et al., 2016), polyclonal goat anti-PAX2 (1:500, AF3364, R&D Systems) and polyclonal rabbit anti-Tyrosine Hydroxylase (TH) (1:1000, AB152, Millipore). Secondary antibodies: donkey anti-rabbit Alexa Fluor 647 (1:1000, AB_2492288, Jackson ImmunoReserch), donkey anti-goat DyLight 405 (1:500, AB_2340426, Jackson ImmunoReserch), and donkey anti-mouse Alexa Fluor 488 (1,1,000 for spinal cord and 1:200 for DRG, A-21202, Thermo Fisher Scientific). Immunofluorescent images were obtained with a confocal laser microscope (LSM780, Carl Zeiss). For quantification of fluorescent intensity of α_2_δ-1 in the spinal dorsal horn and the DRG, 3 images from each mouse were obtained using a 20x objective. We analyzed the fluorescent intensities of α_2_δ-1 in each mCherry-positive neurons’ cell body circled by 12 μm-diameter circular ROI in superficial spinal dorsal horn (within 200 μm depth from the superficial border between white and gray matter including lamina I–III (Sengul, 2013)) using Fiji^1^ and averaged them in each slice. For the α_2_δ-1 quantification in the DRG, we analyzed averaged immunofluorescent intensity of the L4 DRG neurons. We regarded the mean value of 3 slices as the fluorescent intensity of the individual mice.

### RNAscope *in situ* hybridization

RNAscope *in situ* hybridization was performed by the method described previously (Kohro et al., 2020). L3–L4 spinal cord was removed in the same manner described for IHC. Tissues were sectioned at a thickness of 20 μm. Fluorescence *in situ* hybridization (ACDbio) was performed following the manufacturer’s instructions for fixed frozen tissue. The following probes were used: Mm-Cacna2d1 (417141), Mm-Cacna2d2 (449221), Mm-Cacna2d3-C2 (481801-C2) Mm-Slc32a1-C2 (319191-C2), Mm-Slc17a6-C2 (319171-C2), Mm-Slc32a1-C3 (319191-C3), and Mm-Slc17a6-C3 (319171-C3). The superficial SDH regions [within 200 μm depth from the superficial border between white and gray matter including lamina I–III (Sengul, 2013)] in tissue sections were analyzed using LSM780 Imaging System (ZEN 2012, Carl Zeiss). Cells were considered positive if three or more punctate dots were present in the nucleus and/or cytoplasm.

### PNI model

We used the spinal nerve injury model with some modifications (Ho Kim and Mo Chung, 1992; Kohro et al., 2020). The mice were deeply anesthetized with 0.3 mg/kg medetomidine hydrochloride, 4 mg/kg midazolam, and 5 mg/kg butorphanol, and a small incision was made at L3–S1. The paraspinal muscle and fat were removed from the L5 transverse process, which exposed the parallel-lying L3 and L4 spinal nerves. The L4 nerve was then carefully isolated and cut for PNI model, or was left intact for sham model. The wound and the surrounding skin were sutured with cotton thread.

### Behavioral analysis

To assess mechanical hypersensitivity, mice were placed individually in an opaque acrylic box (6 × 6 × 6 cm) on a wire mesh and habituated for ~1 h to allow acclimatization to the experimental environment. Calibrated von Frey filaments (0.02–2.0 g, North Coast Medical) were then applied to the plantar surfaces of the hindpaws of mice from below the mesh floor, and the 50% paw withdrawal threshold was determined using the up–down method (Chaplan et al., 1994; Kohro et al., 2020). von Frey test was carried out before PNI and at 0, 1, 3, 5, 7, 10, 14, 21 and 28 days after PNI.

For the hot-plate test, mice were placed on a metal surface maintained at 52°C in a clear acrylic cylinder (15 cm diameter, 30 cm height) using hot plate equipment (ND1, AS ONE). The latency to either lick the hindpaw or jump was measured as a nocifensive end point, in accordance with previous study (Masuda et al., 2016).

In the formalin-induced pain test, mice were allowed to habituate to the testing environment for ~15 min. Then, mice were injected intraplantarly with formalin (5%, 20 μl), and then the duration of the licking and biting responses to the injected hindpaw was measured at 5 min intervals for 60 min after the injection, as per the previous study (Masuda et al., 2016).

### Slice patch-clamp recordings

We used the methods described in our previous study with some modifications (Yang et al., 2001; Koga et al., 2017). The mice were deeply anesthetized with 0.3 mg/kg medetomidine hydrochloride, 4 mg/kg midazolam, and 5 mg/kg butorphanol, and the lumbosacral spinal cord was removed and placed into a cold pre-oxygenated high sucrose artificial cerebrospinal fluid containing 250 mM sucrose, 2.5 mM KCl, 2 mM CaCl_2_, 2 mM MgCl_2_, 1.2 mM NaH_2_PO_4_, 25 mM NaHCO_3_, and 11 mM glucose. A parasagittal spinal cord slice (250–300 μm thick) with or without L4 dorsal root was made using a vibrating microtome (NLS-MT; Dosaka), and the slice was kept in an artificial cerebrospinal fluid solution (aCSF) containing 125 mM NaCl, 2.5 mM KCl, 2 mM CaCl_2_, 1 mM MgCl_2_, 1.25 mM NaH_2_PO_4_, 26 mM NaHCO_3_, and 20 mM glucose at room temperature (22–25°C) for at least 30 min. The spinal cord slice was then placed into a recording chamber which was continuously superfused with aCSF solution at 27–30°C at a flow rate of 4–6 ml/min. We used two types of internal solutions (Sonohata et al., 2004). The patch pipettes were filled with internal solution containing 110 mM Cs_2_SO_4_, 5 mM tetraethylammonium (TEA), 0.5 mM CaCl_2_, 2 mM MgCl_2_, 5 mM EGTA, 5 mM HEPES, 5 mM QX-314-Br, and 5 mM ATP-Mg (PH 7.2) for excitatory postsynaptic current (EPSC) and inhibitory postsynaptic current (IPSC) recording from single neurons. Internal solution containing 135 mM K-gluconate, 0.5 mM CaCl_2_, 2 mM MgCl_2_, 5 mM KCl, 5 mM EGTA, 5 mM Mg-ATP, and HEPES (pH 7.2 adjusted with KOH) was used to examine the effect of mirogabalin or inhibitory neurotransmitter antagonists on EPSCs. Whole-cell patch-clamp recordings were made from lamina II neurons using MultiClamp 700A amplifier and pCLAMP 10.4 acquisition software (Molecular Devices). The data were digitized with an analog-to-digital converter (Digidata 1321A; Molecular Devices), stored on a computer using a data acquisition program (ClampeX version 8.2; Molecular Devices), and analyzed using a software package (Clampfit version10.4; Molecular Devices). Evoked EPSCs (eEPSCs) and evoked IPSCs (eIPSCs) were induced by a focal monopolar silver electrode (50 μm diameter) insulated except for the tip and placed within ~500 μm from recorded neurons with same stimulation intensity (100 μs, 1.5–2.0 times the threshold required to evoke EPSCs of each neuron) and were isolated and recorded under voltage-clamp conditions at a holding potential of −70 mV or 0 mV, respectively (Xie et al., 2012). For dorsal root stimulation, the dorsal root was stimulated with a suction electrode in A fiber strength [<100 μA for 200 μs (Duan et al., 2014, Francois et al., 2017), 0.05 Hz]. A fiber-evoked responses were considered monosynaptic if the latency remained constant when the root was stimulated at 20 Hz and there was no failure regardless of the constancy of the latency (Baba et al., 2003). We analyzed the amplitude of A fiber-evoked EPSCs in recorded neurons receiving A fiber-evoked mono- or polysynaptic inputs. The EPSC and IPSC areas were quantified by measuring the integrated area (0–100 ms after stimulation) of the synaptic currents. The excitatory and inhibitory ratio was calculated by dividing the evoked EPSC amplitude by the IPSC amplitude evoked by the same intensity stimulation in single neurons. Miniature EPSCs (mEPSCs) were recorded in the presence of TTX for 3 min, and the amplitude and frequency were analyzed with MiniAnalysis software (Synaptosoft). Drugs used were mirogabalin besilate (20 μM, gifted from Daiichi-Sankyo Co., Ltd.), (−)-bicuculline methiodide (10 μM for focal spinal cord stimulation and 20 μM for dorsal root stimulation, 14,343, Fluka Analytical), strychnine hydrochloride hydrate (1 μM for focal spinal cord stimulation and 2 μM for dorsal root stimulation, S0257, Tokyo Chemical Industry), ω-conotoxin GVIA (1 μM, 4,161-v, Peptide Institute, Inc.), ω-agatoxin IVA (200 nM, 4,256-s, Peptide Institute, Inc.), and TTX (0.5 μM, FUJIFILM Wako Chemicals). In the PNI model study, we used the mice 6–10 days after PNI and age-matched naïve cohort mice.

### Statistical analysis

Mice were randomly assigned to each experimental group. For the various analyses, we repeated the experiment in multiple cohorts of animals. In the figure legends, we provide details of sample numbers, statistical tests used and the results of all statistical analyses for each experiment and for all statistical comparisons. Statistical analyses were performed using Prism 9 (GraphPad). All data are shown as the mean ± SEM. Statistical significance of differences was determined using with two-way repeated measures ANOVA with Bonferroni’s multiple comparisons test, two-way ANOVA with Bonferroni’s multiple comparisons test, paired *t*-test, unpaired *t*-test, and unpaired *t*-test with Welch’s correction. Differences were considered significant at *p* < 0.05.

## Results

### α_2_δ-1 mRNAs were selectively expressed in the SDH excitatory neurons

**Figure 1:**
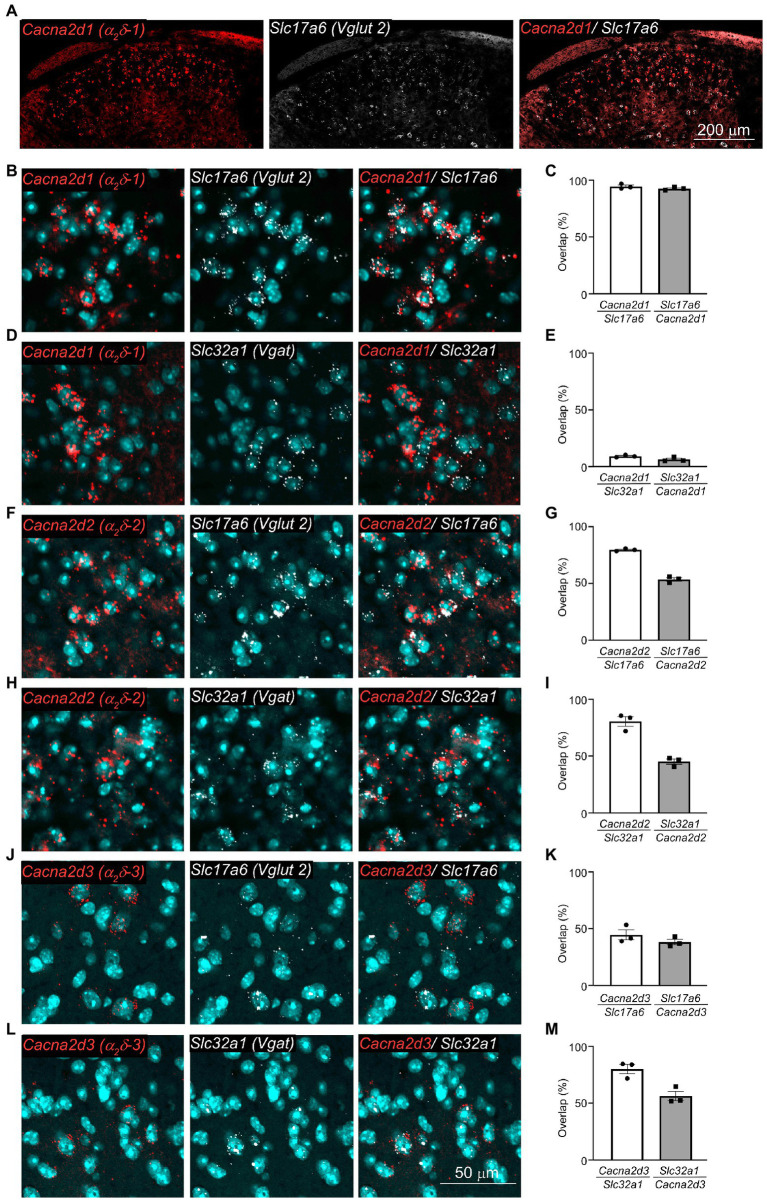
α_2_δ-1 mRNA was selectively expressed in excitatory neurons in the SDH. **(A,B)** Representative images of RNA scope *in situ* hybridization for mRNAs of *Cacna2d1* (encoding α_2_δ-1 subunit, red) and *Slc17a6* (*Vglut2*, gray) in the SDH of naïve mice. Low magnification images **(A)** and high magnification images **(B)**. **(C)** Summary of coexpression of *Cacna2d1* and *Slc17a6*. *n* = 3, 3 sections from 3 animals. **(D)** Representative images of *Cacna2d1* (red) and *Slc32a1* (*Vgat*, gray) mRNAs. **(E)** Summary of coexpression of *Cacna2d1* and *Slc32a1*. *n* = 3, 3 sections from 3 animals. **(F)** Representative images of *Cacna2d2* (encoding α_2_δ-2 subunit, red) and *Slc17a6* (gray) mRNAs. **(G)** Summary of coexpression of *Cacna2d2* and *Slc17a6*. n = 3 sections from 3 animal. **(H)** Representative images of *Cacna2d2* (red) and *Slc32a1* (gray) mRNAs. **(I)** Summary of coexpression of *Cacna2d2* and *Slc32a1*. *n* = 3, 2–3 sections from 3 animals. **(J)** Representative images of *Cacna2d3* (encoding α_2_δ-3 subunit, red) and *Slc17a6* (gray) mRNAs. **(K)** Summary of coexpression of *Cacna2d3* and *Slc17a6*. *n* = 3, 2–3 sections from 3 animals. **(L)** Representative images of *Cacna2d3* (red) and *Slc32a1* (gray) mRNAs. **(M)** Summary of coexpression of *Cacna2d3* and *Slc32a1*. *n* = 3, 2–3 sections from 3 animals. Data are mean ± SEM of averaged data from individual animals.

To explore which types of the spinal excitatory or inhibitory neurons express α_2_δ-1 mRNAs in the SDH, we performed the RNA-scope *in situ hybridization* experiment. As previously reported (Cole et al., 2005), α_2_δ-1 mRNA (*Cacna2d1*) was expressed in the superficial SDH (Figure 1A), and we found that α_2_δ-1 mRNA was mainly detected in excitatory neurons (expressing *Slc17a6* also known as *Vglut2*) (Figures 1A–C, *Cacna2d1/Slc17a6*, 94.6 ± 1.2; *Slc17a6/Cacna2d1*, 92.7 ± 0.8, n = 3 each), but rarely detected in inhibitory neurons (expressing *Slc32a1*, also known as *Vgat*) (Figures 1D,E, *Cacna2d1/Slc32a1*, 9.14 ± 0.59; *Slc32a1/Cacna2d1*, 6.30 ± 1.06, n = 3 each). We also analyzed mRNA expression patterns of other α_2_δ subunits and found that α_2_δ-2 mRNA (*Cacna2d2*) was detected in both types of neurons (Figures 1F–I; Figure 1G: *Cacna2d2/Slc17a6*, 79.9 ± 0.7; *Slc17a6/Cacna2d2*, 53.9 ± 1.6; *n* = 3 each, Figure 1I: *Cacna2d2/Slc32a1*, 80.4 ± 4.3; *Slc32a1/Cacna2d2*, 45.1 ± 2.3, *n* = 3 each), and α_2_δ-3 mRNA (*Cacna2d3*) was also detected in both types of neurons and more frequently observed in inhibitory neurons (Figures 1J–M; Figure 1K: *Cacna2d3/Slc17a6*, 44.6 ± 4.4; *Slc17a6/Cacna2d3*, 38.1 ± 2.4; *n* = 3 each, Figure 1M: *Cacna2d3/Slc32a1*, 79.9 ± 4.2; *Slc32a1/Cacna2d3*, 56.2 ± 4.1, *n* = 3 each). These findings suggest that only α_2_δ-1 mRNA among the three α_2_δ subunits is selectively expressed in excitatory neurons, but not in inhibitory neurons in the SDH.

### α_2_δ-1 in the SDH neurons was involved in mechanical hypersensitivity after PNI

**Figure 2:**
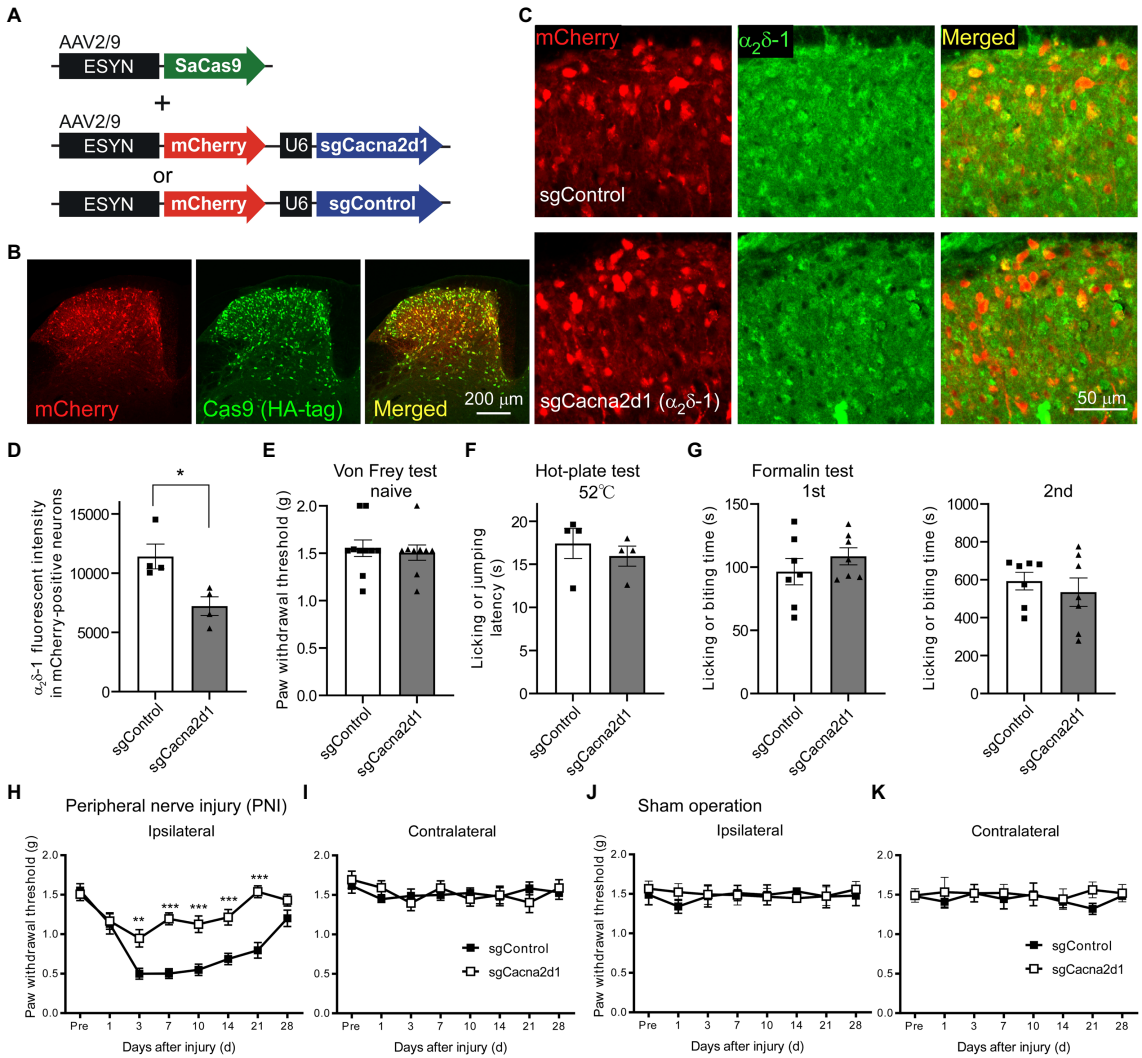
Mechanical hypersensitivity after PNI was ameliorated by AAV-CRISPR-Cas9-mediated α_2_δ-1 knockdown in SDH neurons. **(A)** Schematic of an AAV vector containing a neuronal SaCas9 expression cassette, and vectors containing the sgRNA targeting *Cacna2d1* or control sgRNA fused with a neuronal mCherry expression cassette. **(B)** Representative images showing expression of mCherry (red) and SaCas9 (green) in the SDH. **(C)** Representative images showing α_2_δ-1 expression (green) in mice injected with the Cas9 expression vector and sgControl (*upper*) or sgCacna2d1 (*lower*) containing vector, respectively. **(D)** Summary of the α_2_δ-1 immunofluorescent intensity of mCherry-positive neurons in sgCacna2d1 and sgControl mice (*n* = 4, 3 sections from 4 animals, and data are presented as mean ± SEM of averaged data from individual animals, two-tailed unpaired *t*-test, *t* = 3.12, df = 6, *p < 0.05). **(E)** Summary data of the paw withdrawal threshold of naïve mice [*n* = 10 (sgControl), *n* = 9 (sgCacna2d1), two-tailed unpaired *t*-test, *t* = 0.391, df = 17, *p* = 0.701]. **(F)** Summary data of the latency for mice to lick their hindpaws or jump after being placed on a 52°C hot plate (*n* = 4 each, two-tailed unpaired *t*-test, *t* = 0.692, df = 6, *p* = 0.515). **(G)** Summary of the duration for mice to lick their formalin-injected hindpaw for 0–5 min (first phase, *left*, *n* = 7 each, two-tailed unpaired *t*-test, *t* = 0.984, df = 12, *p* = 0.344) and for 10–60 min (second phase, *right*, *n* = 7 each, two-tailed unpaired *t*-test, *t* = 0.663, df = 12, *p* = 0.520) after formalin injection. **(H)** Paw withdrawal threshold ipsilateral to the injured side of sgControl or sgCacna2d1 mice before (pre) and after PNI. [*n* = 10 (sgControl), *n* = 9 (sgCacna2d1), two-way repeated measurements ANOVA post-hoc Bonferroni’s test, Group, *F*_(1,17)_ = 46.1, *p* < 0.0001, Time, *F*_(7,119)_ = 20.2, *p* < 0.0001, Interaction *F*_(7,119)_ = 5.91, *p* < 0.0001, **p < 0.01, ***p < 0.001]. **(I)** Same as **(H)** but contralateral to the injured side [*n* = 10 (sgControl), *n* = 9 (sgCacna2d1), two-way repeated measurements ANOVA *post hoc* Bonferroni’s test, Group, *F*_(1,17)_ = 0.00552, *p* = 0.942, Time, *F*_(7,119)_ = 1.07, *p =* 0.386, Interaction *F*_(7,119)_ = 0.788, *p* = 0.599]. **(J)** Paw withdrawal threshold ipsilateral to the injured side of sgControl or sgCacna2d1 mice before (pre) and after sham operation [*n* = 6 each, two-way repeated measurements ANOVA post-hoc Bonferroni’s test, Group, *F*_(1,10)_ = 0205, *p* = 0.660, Time, *F*_(7,70)_ = 0.155, *p =* 0.993, Interaction *F*_(7,70)_ = 0.293, *p* = 0.955]. **(K)** Same as **(J)** but contralateral to the operated side [*n* = 6 each, two-way repeated measurements ANOVA post-hoc Bonferroni’s test, Group, *F*_(1,17)_ = 0.894, *p* = 0.367, Time, *F*_(7,70)_ = 1.83, *p =* 0.988, Interaction *F*_(7,70)_ = 0.356, *p* = 0.924]. Data are mean ± SEM.

To determine the functional role of α_2_δ-1 in the SDH neurons, we utilized AAV-CRISPR-Cas9-mediated *in vivo* genome editing techniques (Ran et al., 2015). We injected an AAV-ESYN-SaCas9 vector, which enabled neuronal expression of SaCas9, and AAV vectors containing a *Cacna2d1*-targeting single-guide RNA (sgCacna2d1) cassette, or a control sgRNA cassette (sgControl) fused with ESYN-mCherry cassette into the SDH (Figure 2A). Six weeks after microinjection, mCherry and SaCas9 (labeled by HA-tag) were expressed in the SDH neurons (Figure 2B; Supplementary Figure S1), and 67.2 ± 2.5% and 66.4 ± 2.6% of the NeuN positive neurons were also HA-tag- and mCherry-positive in sgControl and sgCacna2d1 mice, respectively (*n* = 4 each). On the other hand, we did not observe any mCherry expression in both the L4 DRG and the brain regions sending inputs to the spinal cord (Supplementary Figure S2). In the sgCacna2d1 mice, α_2_δ-1 immunoreactivity was significantly decreased in SDH mCherry-expressing neurons compared to that in the sgControl mice (Figures 2C,D, sgControl, 11,411 ± 1,046; sgCacna, 7,221 ± 788, *n* = 4 each). To investigate the behavioral role of spinal α_2_δ-1 for acute pain, we performed von Frey, hot-plate, and formalin test using these mice. In naïve conditions, behavioral responses to mechanical stimuli, heat and formalin administration were indistinguishable between sgCacna2d1 and sgControl groups [Figures 2E–G; Figure 2E: sgControl (*n* = 10), 1.55 ± 0.09 g; sgCacna (*n* = 9), 1.51 ± 0.08 g, Figure 2F: sgControl (*n* = 4), 17.4 ± 1.7 s; sgCacna (*n* = 4), 16.0 ± 1.2 s, Figure 2G: first phase, *left*: sgControl (*n* = 7), 96.4 ± 10.3 s; sgCacna (*n* = 7), 108.6 ± 6.8 s, second phase, *right*: sgControl (*n* = 7), 593.0 ± 46.6 s; sgCacna (*n* = 7), 534.4 ± 75.0 s]. As α_2_δ-1 subunit is the main target of gabapentinoids, drugs used for neuropathic pain treatment (Taylor, 2009), we investigated whether spinal α_2_δ-1 contributes to mechanical hypersensitivity after PNI. We found that decrease of paw withdrawal threshold (mechanical hypersensitivity) after PNI was ameliorated in sgCacna2d1 mice 3–21 days after PNI (Figures 2H,I), while there is no significant difference between two groups in the upregulation of α_2_δ-1 in the DRG after PNI [Supplementary Figure S3, sgControl (naïve), 3,850 ± 81; sgControl (PNI), 7,359 ± 349; sgCacna2d1 (naïve), 4,050 ± 180; sgCacna2d1 (PNI), 7,077 ± 328, *n* = 5 each]. In sham-operated mice, we did not observe any significant difference in paw withdrawal threshold between two groups (Figures 2J,K). These findings suggest that α_2_δ-1 in the SDH neurons is involved in mechanical hypersensitivity after PNI, but not in basal mechanical and acute pain sensitivity.

### EPSCs and IPSCs in the SDH neurons were increased or decreased, respectively, after PNI

**Figure 3:**
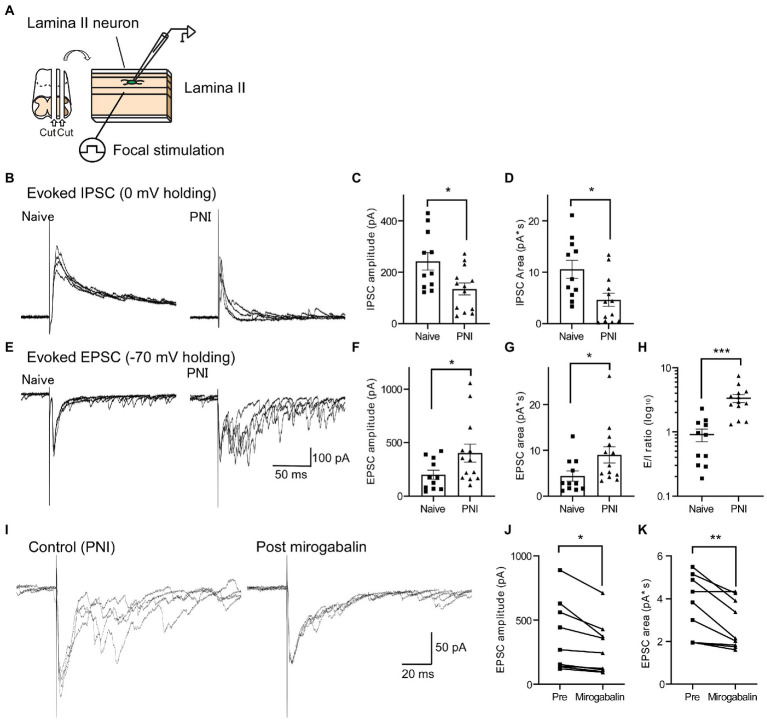
EPSCs and IPSCs in SDH neurons were increased or decreased, respectively, after PNI. **(A)** Schematic of the whole-cell patch-clamp recording from SDH neurons and electrical focal SDH stimulation. **(B)** Representative traces of evoked IPSCs in SDH neurons of naïve (left) or PNI (day 6–10, right) mice. **(C,D)** Summary of IPSC amplitude of naïve and PNI mice [**C**; *n* = 11 (naïve), *n* = 13 (PNI), unpaired *t*-test, *t* = 2.69, df = 22, *p < 0.05] and IPSC area [**D**; *n* = 11 (naïve), *n* = 13 (PNI), unpaired *t*-test, *t* = 2.79, df = 22, *p < 0.05]. **(E–G)** Same as **(B–D)** but those of evoked EPSC in the same recorded neurons. Representative traces **(E)**, EPSC amplitude [**F**; *n* = 11 (naïve), *n* = 13 (PNI), unpaired *t*-test with Welch’s correction, *t* = 2.15, df = 17.6, *p < 0.05] and EPSC area [**G**; *n* = 11 (naïve), *n* = 13 (PNI), unpaired *t*-test, t = 2.11, df = 22, *p < 0.05]. **(H)** Summary of E/I ratio of naïve and PNI mice [*n* = 11 (naïve), *n* = 13 (PNI), unpaired *t*-test with Welch’s correction, *t* = 4.66, df = 16.0, ***p < 0.001]. **(I)** Representative traces showing the effect of mirogabalin, a potent α_2_δ-1 subunit ligand, on enhanced eEPSCs after PNI. **(J,K)** Summary showing the effect of mirogabalin on eEPSC amplitude (**J**: *n* = 9, paired *t*-test, *t* = 3.19, df = 8, *p < 0.05) and area (**K**: *n* = 9, paired *t*-test, *t* = 3.61, df = 8, **p < 0.01). Data are mean ± SEM.

To examine the alternation of synaptic transmission in SDH neurons after PNI, we performed slice patch-clamp recording from lamina II neurons and recorded both IPSCs and EPSCs evoked by electrical stimulation applied to deeper layer of the SDH from each single SDH neuron at holding potential of 0 mV or −70 mV, respectively (Figure 3A). We found that while the IPSC amplitude and IPSC area were decreased 6–10 days after PNI [Figures 3B–D; Figure 3C: naïve (*n* = 11), 243.0 ± 34.0 pA; PNI (*n* = 13), 134.8 ± 23.3 pA, Figure 3D: naïve (*n* = 11), 10.58 ± 1.76 pA*s; PNI (*n* = 13), 4.63 ± 1.27 pA*s], the EPSC amplitude and EPSC area were increased after PNI [Figures 3E–G; Figure 3F: naïve (*n* = 11), 198.8 ± 42.7 pA; PNI (*n* = 13), 401.2 ± 84.1 pA, Figure 3G: naïve (*n* = 11), 4.35 ± 1.12 pA*s; PNI (*n* = 13), 8.98 ± 1.77 pA*s]. The excitatory and inhibitory ratio (E/I ratio) of single SDH neurons was significantly increased after PNI [Figure 3H, naïve (*n* = 11), 0.91 ± 0.20; PNI (*n* = 13), 3.35 ± 0.48]. These evoked EPSCs after PNI were almost completely blocked by co-application of ω-agatoxin IVA (200 nM) and ω-conotoxin GVIA (1 μM), inhibitor of P/Q-type and N-type calcium channels which contain α_2_δ-1 subunits (pre-drug, 385.8 ± 127.8 pA; post-drug, 33.4 ± 10.7 pA; n = 5, *p* < 0.05, two-tailed paired *t*-test), suggesting involvement of VGCCs in this transmission. To investigate the contribution of α_2_δ-1 to the aberrant excitability after PNI, we used a potent α_2_δ-1 ligand, mirogabalin (Kitano et al., 2019). Mirogabalin application (20 μM) significantly decreased the enhanced EPSC amplitude and area after PNI (Figures 3I–K; Figure 3J: pre, 371.8 ± 91.7 pA; mirogabalin, 283.0 ± 69.2 pA, Figure 3K: pre, 3.61 ± 0.48 pA*s; mirogabalin, 2.81 ± 0.38 pA*s, *n* = 9 each). These findings suggest that α_2_δ-1 is involved in aberrant excitatory transmission in the SDH after PNI. As it has been reported that gabapentinoids affect VGCCs activity (Biggs et al., 2014) and modulate mEPSCs and sEPSCs in PNI model mice (Matsuzawa et al., 2014; Alles et al., 2017), we recorded mEPSC in the presence of TTX in PNI mice and investigated whether mirogabalin application affects the mEPSC frequency and amplitude. In consistent with the previous report (Matsuzawa et al., 2014), mirogabalin bath-application significantly reduced the mEPSC frequency (Supplementary Figure S4A, pre: 11.7 ± 2.5 Hz, mirogabalin: 10.4 ± 2.1 Hz, *n* = 14, two-tailed paired *t-*test, *p* = 0.043) but did not significantly affect the mEPSC amplitude (Supplementary Figure S4B, pre: 16.2 ± 1.1 pA, mirogabalin: 15.6 ± 0.9 pA, n = 14, two-tailed paired *t-*test, *p* = 0.112), suggesting mirogabalin presynaptically affects the excitatory synaptic transmission in SDH neurons *via* the terminals of spinal dorsal horn neurons and/or primary afferents.

As the spinal dorsal horn contains both excitatory and inhibitory neurons, our analysis could include recordings from both excitatory and inhibitory interneurons. However, because more than half of neurons (~70%) in lamina II were excitatory (Todd, 2010), we assumed that our recording results are likely to reflect excitatory synaptic facilitation in the excitatory neurons. However, it was reported that excitatory synaptic transmissions and excitability in inhibitory neurons were decreased after nerve injury (Leitner et al., 2013; Duan et al., 2014). Therefore, using inhibitory neuron-specific Cre-expressing *Vgat-Cre* mice (Vong et al., 2011) and a Cre-induced mCherry expression vector (AAV9-EF1α-FLEX-mCherry), we fluorescently labeled SDH inhibitory neurons (Supplementary Figures S5A,B) and investigated synaptic transmission in the SDH inhibitory neurons after PNI. Although the amplitude and area of IPSCs evoked by focal stimulation in the mCherry-labeled neurons were indistinguishable between naïve and PNI groups [Supplementary Figures S5C–E; Supplementary Figure S5D: naïve (*n* = 15), 229.7 ± 35.0 pA; PNI (*n* = 16), 265.2 ± 47.5 pA, Supplementary Figure S5E: naïve (*n* = 15), 9.79 ± 1.74 pA*s; PNI (*n* = 16), 8.44 ± 1.49 pA*s], the evoked EPSC amplitude tended to be decreased and the evoked EPSC area was significantly reduced after PNI in the mCherry-labeled neurons [Supplementary Figures S5F–I; Supplementary Figure S5G: naïve (*n* = 15), 377.9 ± 81.3 pA; PNI (*n* = 16), 223.9 ± 36.1 pA, Supplementary Figure S5H: naïve (*n* = 15), 6.06 ± 1.13 pA*s; PNI (*n* = 16), 2.98 ± 0.53 pA*s]. On the other hand, the E/I ratio of mCherry-labeled neurons was not significantly changed after PNI [Supplementary Figure S5I, naïve (*n* = 15), 2.17 ± 0.56; PNI (*n* = 16), 1.32 ± 0.24]. These data suggest that synaptic transmission in inhibitory and excitatory neurons might be differently modulated after nerve injury.

### Spinal α_2_δ-1 subunits were involved in facilitation of EPSCs after PNI

**Figure 4:**
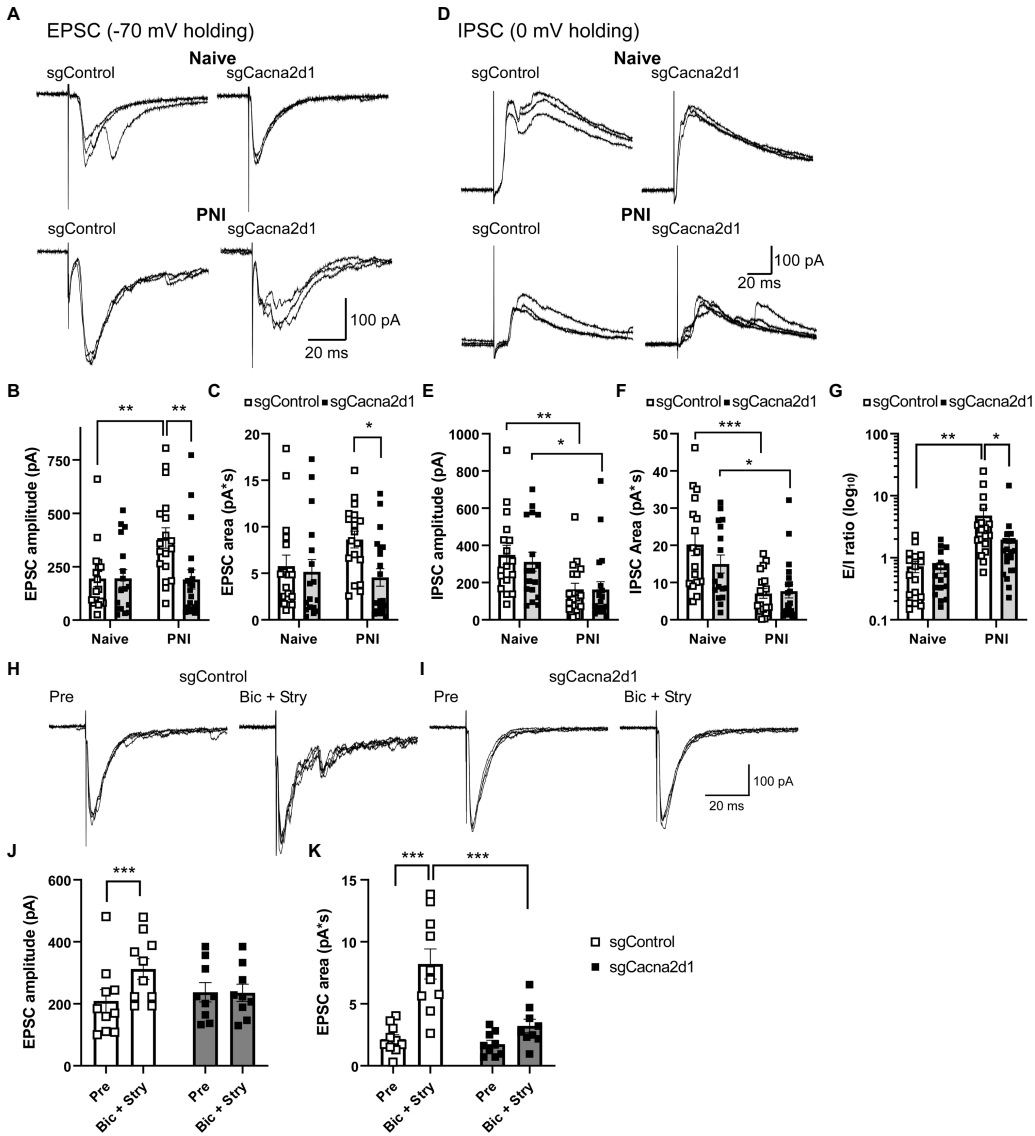
α_2_δ-1 in SDH neurons was involved in disinhibition-induced EPSC facilitation after PNI. **(A)** Representative traces showing eEPSCs in SDH neurons of sgControl (*left traces*) or sgCacna2d1 mice (*right traces*) without (*upper traces*) or with PNI (*lower traces*). **(B,C)** Summary showing eEPSC amplitude [**B**: *n* = 17 (naïve, sgControl), *n* = 17 (naïve, sgCacna2d1), *n* = 18 (PNI, sgControl), *n* = 20 (PNI, sgCacna2d1), two-way ANOVA *post hoc* Bonferroni’s test, Group, *F*_(1,68)_ = 4.93, *p* = 0.030, Treatment, *F*_(1,68)_ = 4.52, *p* = 0.037, Interaction *F*_(1,68)_ = 5.04, *p* = 0.028, **p < 0.01] and eEPSC area [**C**: *n* = 17 (naïve, sgControl), *n* = 17 (naïve, sgCacna2d1), *n* = 18 (PNI, sgControl), *n* = 20 (PNI, sgCacna2d1), two-way ANOVA *post hoc* Bonferroni’s test, Group, *F*_(1,68)_ = 4.61, *p* = 0.035, Treatment, *F*_(1,68)_ = 1.06, *p* = 0.306, Interaction *F*_(1,68)_ = 2.54, *p* = 0.116, *p < 0.05]. **(D)** Representative traces showing eIPSCs in SDH neurons of sgControl (*left*) or sgCacna2d1 mice (*right*) without (*upper*) or with PNI (*lower*). **(E,F)** Summary showing eIPSC amplitude [**E**: *n* = 17 (naïve, sgControl), *n* = 17 (naïve, sgCacna2d1), *n* = 18 (PNI, sgControl), *n* = 20 (PNI, sgCacna2d1), two-way ANOVA *post hoc* Bonferroni’s test, Group, *F*_(1,68)_ = 0.181, *p* = 0.672, Treatment, *F*_(1,68)_ = 14.0, *p* = 0.0004, Interaction *F*_(1,68)_ = 0.168, *p* = 0.684, *p < 0.05*,* **p < 0.01], and eIPSC area [**F**: *n* = 17 (naïve, sgControl), *n* = 17 (naïve, sgCacna2d1), *n* = 18 (PNI, sgControl), *n* = 20 (PNI, sgCacna2d1), two-way ANOVA *post hoc* Bonferroni’s test, Group, *F*_(1,68)_ = 1.14, *p* = 0.289, Treatment, *F*_(1,68)_ = 22.0, *p <* 0.0001, Interaction *F*_(1, 68)_ = 1.79, *p* = 0.185, *p < 0.05, ***p < 0.001]. **(G)** Summary of E/I ratio of each group. [*n* = 17 (naïve, sgControl), *n* = 17 (naïve, sgCacna2d1), *n* = 18 (PNI, sgControl), *n* = 20 (PNI, sgCacna2d1), two-way ANOVA *post hoc* Bonferroni’s test, Group, *F*_(1,68)_ = 2.99, *p* = 0.089, Treatment, *F*_(1,68)_ = 10.3, *p* = 0.002, Interaction *F*_(1,68)_ = 2.87, *p* = 0.095, *p < 0.05, **p < 0.01]. **(H,I)** Representative traces showing eEPSCs in SDH neurons of sgControl **(H)** or sgCacna2d1 **(I)** mice before (*left*) and after bicuculine (10 μM) and strychnine (1 μM) application (Bic + Stry, *right*). **(J,K)** Summary showing eEPSC amplitude of each group [**J**: *n* = 10 (sgControl), *n* = 9 (sgCacna2d1), two-way repeated measurements ANOVA *post hoc* Bonferroni’s test, Group, *F*_(1,17)_ = 0.319, *p* = 0.580, Treatment, *F*_(1,17)_ = 9.59, *p* = 0.007, Interaction *F*_(1,17)_ = 10.3, *p* = 0.005, ***p < 0.001] and eEPSC area of each group [**K**: *n* = 10 (sgControl), *n* = 9 (sgCacna2d1), two-way repeated measurements ANOVA *post hoc* Bonferroni’s test, Group, *F*_(1,17)_ = 11.5, *p* = 0.003, Treatment, *F*_(1,17)_ = 32.4, *p* < 0.0001, Interaction *F*_(1,17)_ = 11.2, *p* = 0.003, ***p < 0.001]. Data are mean ± SEM.

To further investigate whether α_2_δ-1 in SDH neurons involved in the altered synaptic transmission after PNI, we used AAV-CRISPR-Cas9 mediated SDH α_2_δ-1 knockdown mice as shown in Figure 2. Basal EPSC amplitude and area were indistinguishable between sgControl and sgCacna2d1 groups in naïve mice; however, after PNI, only sgControl groups showed significantly enhanced EPSC amplitude and area, while the enhancement in sgCacna2d1 group was much less pronounced [Figures 4A–C; Figure 4B: sgControl (naïve) (*n* = 17), 194.7 ± 37.4 pA; sgCacna2d1 (naïve) (*n* = 17), 195.8 ± 40.2 pA; sgControl (PNI) (*n* = 18), 384.3 ± 47.8 pA; sgCacna2d1 (PNI) (*n* = 20), 190.6 ± 45.1 pA, Figure 4C: sgControl (naïve) (*n* = 17), 5.77 ± 1.20 pA*s; sgCacna2d1 (naïve) (*n* = 17), 5.17 ± 1.31 pA*s; sgControl (PNI) (*n* = 18), 8.62 ± 0.89 pA*s; sgCacna2d1 (PNI) (*n* = 20), 4.55 ± 0.95 pA*s]. On the other hand, basal IPSC amplitude and area in naïve mice, and decreases in IPSC amplitude and area after PNI were very similar between sgCacna2d1 and sgControl groups [Figures 4D–F; Figure 4E: sgControl (naïve) (*n* = 17), 348.7 ± 51.8 pA; sgCacna2d1 (naïve) (*n* = 17), 311.6 ± 51.0 pA; sgControl (PNI) (*n* = 18), 163.9 ± 31.9 pA; sgCacna2d1 (PNI) (*n* = 20), 163.3 ± 41.7 pA, Figure 4F: sgControl (naïve) (*n* = 17), 20.21 ± 2.97 pA*s; sgCacna2d1 (naïve) (*n* = 17), 14.96 ± 2.42 pA*s; sgControl (PNI) (*n* = 18), 7.08 ± 1.31 pA*s; sgCacna2d1 (PNI) (*n* = 20), 7.67 ± 1.83 pA*s], suggesting α_2_δ-1 in SDH neurons is not involved in loss of inhibition after PNI. Enhanced E/I ratio after PNI was also ameliorated in sgCacna2d1 group [Figure 4G, sgControl (naïve) (*n* = 17), 0.73 ± 0.15; sgCacna2d1 (naïve) (*n* = 17), 0.70 ± 0.13; sgControl (PNI) (*n* = 18), 4.80 ± 1.46; sgCacna2d1 (PNI) (*n* = 20), 1.96 ± 0.69]. Furthermore, we also analyzed mEPSCs in the presence of TTX and found that the mEPSC frequency in sgControl group after PNI were significantly facilitated, and the facilitation was decreased in sgCacna2d1 group [Supplementary Figures S6A,B, sgControl (naïve) (*n* = 16), 2.82 ± 0.94 Hz; sgCacna2d1 (naïve) (*n* = 12), 1.56 ± 0.34 Hz; sgControl (PNI) (*n* = 17), 11.27 ± 2.21 Hz; sgCacna2d1 (PNI) (*n* = 16), 5.84 ± 1.66 Hz], but there is not significant difference in the mEPSC amplitude between those groups [Supplementary Figure S6C, sgControl (naïve) (*n* = 16), 15.8 ± 1.4 pA; sgCacna2d1 (naïve) (*n* = 12), 12.8 ± 0.6 pA; sgControl (PNI) (*n* = 17), 15.6 ± 0.8 pA; sgCacna2d1 (PNI) (*n* = 16), 14.4 ± 0.8 pA], suggesting α_2_δ-1 in SDH neurons involved in the presynaptic enhancement of glutamate release after nerve injury. These findings suggest that α_2_δ-1 expressed in SDH neurons is involved in the enhanced excitability in the SDH after PNI, but not in decreased inhibitory transmission.

### Spinal α_2_δ-1 subunits were involved in disinhibition-mediated enhancement of excitatory synaptic transmission

Attenuation of inhibitory transmission in the SDH is thought to be one of the important mechanisms of aberrant excitability after PNI (Moore et al., 2002; Baba et al., 2003). Therefore, we examined whether α_2_δ-1 in SDH neurons is involved in disinhibition-induced facilitation of excitatory synaptic transmission using inhibitory neurotransmitter antagonists. Application of bicuculine (10 μM), a GABA_A_ receptor antagonist, and strychnine (1 μM), a glycine receptor antagonist, increased the amplitude and area of the evoked EPSCs in sgControl mice, but in sgCacna2d1 mice the disinhibition-mediated facilitation of the EPSCs was significantly suppressed compared to that of sgControl mice [Figures 4H–K; Figure 4J: sgControl (pre) (*n* = 10), 209.3 ± 36.5 pA; sgControl (bic + stry) (*n* = 10), 312.0 ± 33.3 pA; sgCacna2d1 (pre) (*n* = 9), 237.1 ± 31.1 pA; sgCacna2d1 (bic + stry) (*n* = 9), 235.3 ± 27.8 pA, Figure 4K: sgControl (pre) (*n* = 10), 2.15 ± 0.36 pA*s; sgControl (bic + stry) (*n* = 10), 8.20 ± 1.21 pA*s; sgCacna2d1 (pre) (*n* = 9), 1.74 ± 0.32 pA*s; sgCacna2d1 (bic + stry) (*n* = 9), 3.21 ± 0.55 pA*s]. It is known that A fiber input is strongly modulated in disinhibition condition (Baba et al., 2003; Duan et al., 2014; Peirs et al., 2015). Therefore, we tested whether the A fiber-induced EPSC facilitation by disinhibition also affected by the α_2_δ-1 knock down using spinal cord slices with L4 dorsal root attached (Supplementary Figure S7A). While the basal A fiber-evoked EPSC amplitude and area were indistinguishable between two groups, the A fiber-evoked EPSCs facilitation by application of bicuculine (20 μM) and strychnine (2 μM) observed in sgControl group was significantly attenuated in sgCacna2d1 group [Supplementary Figures S7B–E; Supplementary Figures S7D: sgControl (pre) (*n* = 12), 161.6 ± 23.3 pA; sgControl (bic + stry) (*n* = 12), 326.9 ± 47.4 pA; sgCacna2d1 (pre) (*n* = 19), 175.0 ± 40.2 pA; sgCacna2d1 (bic + stry) (*n* = 19), 209.8 ± 41.1 pA, Supplementary Figures S7E: sgControl (pre) (*n* = 12), 2.71 ± 0.48 pA*s; sgControl (bic + stry) (*n* = 12), 11.38 ± 2.92 pA*s; sgCacna2d1 (pre) (*n* = 19), 2.34 ± 0.58 pA*s; sgCacna2d1 (bic + stry) (*n* = 19), 5.37 ± 1.49 pA*s]. These findings suggest that α_2_δ-1 in SDH neurons is involved in the disinhibition-mediated facilitation of excitatory synaptic transmission.

## Discussion

**Figure 5:**
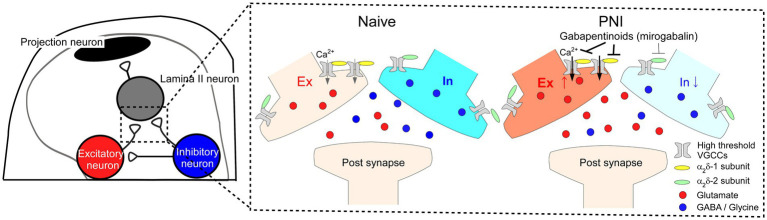
Potential mechanisms of disinhibition-induced enhanced excitatory synaptic transmission *via* α_2_δ-1 expressed in SDH excitatory neurons after PNI and action of gabapentinoids. Schematic of proposed spinal circuitry **(left)** and mechanisms of the alteration of synaptic transmission after PNI and gabapentinoid action **(right)**. In PNI mice, excitatory and inhibitory synaptic transmissions are enhanced and attenuated in SDH neurons, respectively. α_2_δ-1 expressed in excitatory SDH neurons could be involved in the enhanced excitatory neurotransmitter release, and gabapentinoids preferentially bind to α_2_δ-1 in excitatory SDH neurons and inhibit the aberrant excitatory synaptic responses and the pain phenotype after PNI.

In this study, we found that α_2_δ-1 is selectively expressed in excitatory SDH neurons, while α_2_δ-2 and α_2_δ-3 are expressed in both excitatory and inhibitory neurons. Furthermore, using the AAV-CRISPR-Cas9-mediated SDH neuron-specific gene ablation technique, we showed that α_2_δ-1 in SDH neurons is involved in disinhibition-induced facilitation of excitatory synaptic transmission and is important for mechanical hypersensitivity and aberrant excitatory neurotransmission in SDH after PNI (Figure 5). Our findings are crucial for understanding spinal mechanisms of both pain facilitation after nerve injury and pharmacology of gabapentinoids.

Previous studies have reported that α_2_δ-1 in SDH neurons is upregulated after spinal cord injury (SCI) and important for the following mechanical hypersensitivity (Boroujerdi et al., 2011; Kusuyama et al., 2018). It has been also suggested that α_2_δ-1 in the SDH, including SDH neurons and peripheral central terminals, is involved in mechanical hypersensitivity after PNI (Li et al., 2004; Chen et al., 2018), but exact contribution of α_2_δ-1 in SDH neurons to neuropathic pain has not previously been addressed. It is because previous studies used α_2_δ-1 knockout mice or genetic and pharmacological regents delivered intrathecally (Li et al., 2004; Chen et al., 2018), which affect α_2_δ-1 expression or function both in SDH neurons and DRG neurons. In this study, we used AAV-CRISPR-Cas9 system (Ran et al., 2015) and intraspinal microinjection method (Kohro et al., 2015) to selectively manipulate α_2_δ-1 expression in SDH neurons. In our results, we could not detect any mCherry expression in the DRG and the brain regions with spinal projection, but we observed efficient viral transduction and downregulation of α_2_δ-1 in the SDH neurons in the manipulated mice. As these data suggest that our strategy enabled to selectively manipulate α_2_δ-1 expression in SDH neurons, our results indicate that α_2_δ-1 in SDH neurons contribute to the mechanical hypersensitivity and aberrant excitability after PNI. It has been reported that α_2_δ-1 knockout mice exhibit reduction in mechanical and cold sensitivity, and delayed mechanical hypersensitivity after PNI (Patel et al., 2013). However, other studies showed that genetic or pharmacological inhibition of α_2_δ-1 did not induce any such mechanical deficit in normal condition (Dooley et al., 2007; Park et al., 2016), and we did not observe any alteration in mechanical sensitivity in naïve mice with α_2_δ-1 knockdown in SDH neurons, but these manipulations alleviated mechanical hypersensitivity after PNI. These results suggest that developmental α_2_δ-1 expression and/or α_2_δ-1 in other brain regions could be important for basal mechanical and cold sensitivity and regulation of neuropathic pain phenotype after nerve injury.

α_2_δ subunits, especially α_2_δ-1–3, are distributed in the CNS and PNS (Taylor, 2009), and we found that each of these α_2_δ subunits exhibit distinct expression patterns in the SDH. Only α_2_δ-1 mRNA among the three subunits, the main target of gabapentinoids, was selectively observed in excitatory neurons in the SDH, while α_2_δ-2 and α_2_δ-3 mRNAs were observed both in excitatory and inhibitory neurons, which is supported by a recent single cell RNA sequencing study (Haring et al., 2018). Our findings could be one of the reasons why α_2_δ-1, but not α_2_δ-2, is important for mechanical hypersensitivity after PNI and for analgesic effect of gabapentinoids (Dooley et al., 2007). A body of literature suggest that upregulation of α_2_δ-1 in the DRG is important for neuropathic pain phenotype after PNI (Li et al., 2004; Dooley et al., 2007). Furthermore, the mechanical pain phenotype induced by intrathecal injection of thrombospondin-4, known to contributes to neuropathic pain, is not observed in DRG neuron-specific α_2_δ-1 conditional knockout mice (Park et al., 2016). Our study and previous studies suggest that α_2_δ-1 in both SDH neurons and DRG neurons contribute to neuropathic pain phenotypes after PNI.

α_2_δ-1 is known to modulate VGCCs current density and membrane trafficking (Taylor, 2009), and to be involved in the modulation of presynaptic neurotransmitter release after nerve injury (Matsuzawa et al., 2014; Alles et al., 2017; Chen et al., 2018). Our results also suggest that α_2_δ-1 in SDH neurons contribute to the presynaptic modulation in neuropathic pain model, as the facilitated mEPSC frequency after PNI were reduced by the α_2_δ-1 knockdown. In contrast, we did not observe significant differences in the mEPSC amplitude in knockdown animals in this study, which did not support postsynaptic action of α_2_δ-1, but further studies are needed to elucidate the postsynaptic role of α_2_δ-1 in pain facilitation after nerve injury. On the other hand, we found that the IPSC amplitude evoked by focal stimulation was decreased after nerve injury, which was not affected by the α_2_δ-1 knockdown. These electrophysiological results were consistent with the α_2_δ-1 localization in excitatory and inhibitory neurons of the spinal dorsal horn. Both presynaptic and postsynaptic mechanisms have been suggested to be involved in the attenuation of inhibitory neurotransmissions after nerve injury (Moore et al., 2002; Kurabe et al., 2022), but further studies are needed to elucidate the underlying mechanisms.

Loss of inhibition is thought to be one of the important mechanisms of mechanical hypersensitivity and aberrant excitability after PNI or SCI (Moore et al., 2002; Baba et al., 2003; Lu et al., 2008). Although treatment of bicuculine and strychnine is not justified to mimic nerve injury state, this treatment is used to investigate underlying mechanisms of neuropathic pain (Duan et al., 2014; Peirs et al., 2015). Using a PNI model and treatment of bicuculine and strychnine, our data suggest that α_2_δ-1 in SDH neurons is important for disinhibition-mediated aberrant excitability in the SDH, but not for attenuation of inhibitory neurotransmission after PNI. Disinhibition is thought to induce strong and long-lasting depolarization of SDH neurons after stimulation *via* NMDA receptor activation (Baba et al., 2003), that could in turn activate the VGCCs containing α_2_δ-1, and result in further Ca^2+^ influx (D'Arco et al., 2015) and aberrant release of excitatory neurotransmitters. This assumption is supported by previous studies showing that disinhibition-induced mechanical hypersensitivity and spontaneous pain-related behavior are ameliorated by intrathecal administration of gabapentinoids (Tsukamoto et al., 2010; Koga et al., 2017). It is also reported that α_2_δ-1 is coupled with NMDA receptors (Chen et al., 2018), and this coupling could also play a role in disinhibition-mediated pain facilitation.

α_2_δ-1 is widely distributed in the CNS and PNS (Cole et al., 2005), and α_2_δ-1 expressed in the brain is involved in synaptic transmission and pain processing (Taylor and Garrido, 2008; Yamamoto et al., 2021). Further studies are needed to elucidate the exact contribution of α_2_δ-1 in each CNS region to mechanical hypersensitivity or other psychological disorder after PNI, and to the pharmacological action of gabapentinoids.

In summary, we identified that α_2_δ-1 is mainly expressed in excitatory neurons in the SDH, and showed that α_2_δ-1 in SDH neurons contributes to the aberrant excitatory synaptic transmissions and mechanical hypersensitivity after PNI. Our findings are important for further understanding of pharmacological action of gabapentinoids, the commonly used drugs for neuropathic pain, and can be a clue to explain why α_2_δ-1 among α_2_δ subunits is important for pain hypersensitivity after nerve injury.

## Data availability statement

The raw data supporting the conclusions of this article will be made available by the corresponding authors upon reasonable request.

## Author contributions

KeiK conceived the project, designed the experiments, performed almost all experiments, analyzed the data, and wrote the manuscript. KenK and MT provided the critical materials and advised on data interpretation. KaK and YK advised on experimental design. HF conceived and supervised this project, designed the experiments, and edited the manuscript. All authors read and approved the manuscript.

## Funding

This work was supported by following grants, JSPS KAKENHI Grant JP20H04043 (HF), JSPS KAKENHI Grant JP20K16133 (KeiK), JSPS KAKENHI Grant JP22K15206 (KeiK), Hyogo Innovative Challenge grant (HF), Hyogo Medical University Grant for Research Promotion 2021 (KeiK), Takeda Science Foundation (KeiK) and conducted as the collaborative study with Daiichi Sankyo Co., Ltd. Daiichi Sankyo Co., Ltd was not involved in the study design, collection, analysis, interpretation of data, the writing of this article or the decision to submit it for publication.

## Conflict of interest

Kak and YK are employees of Daiichi Sankyo Co., Ltd.

The remaining authors declare that the research was conducted in the absence of any commercial or financial relationships that could be construed as a potential conflict of interest.

## Publisher’s note

All claims expressed in this article are solely those of the authors and do not necessarily represent those of their affiliated organizations, or those of the publisher, the editors and the reviewers. Any product that may be evaluated in this article, or claim that may be made by its manufacturer, is not guaranteed or endorsed by the publisher.

## Footnotes

^1^ http://fiji.sc
