# Supplementary material for: Voltage-gated calcium channel subunit α2δ-1 in spinal dorsal horn neurons contributes to aberrant excitatory synaptic transmission and mechanical hypersensitivity after peripheral nerve injury
Source: Front Mol Neurosci. 2023 Mar 23;16:1099925. doi: 10.3389/fnmol.2023.1099925 (PMC10076860; doi:10.3389/fnmol.2023.1099925)
Supplement: Supplementary file 1 [file Data_Sheet_1.docx]

Supplementary Material

**
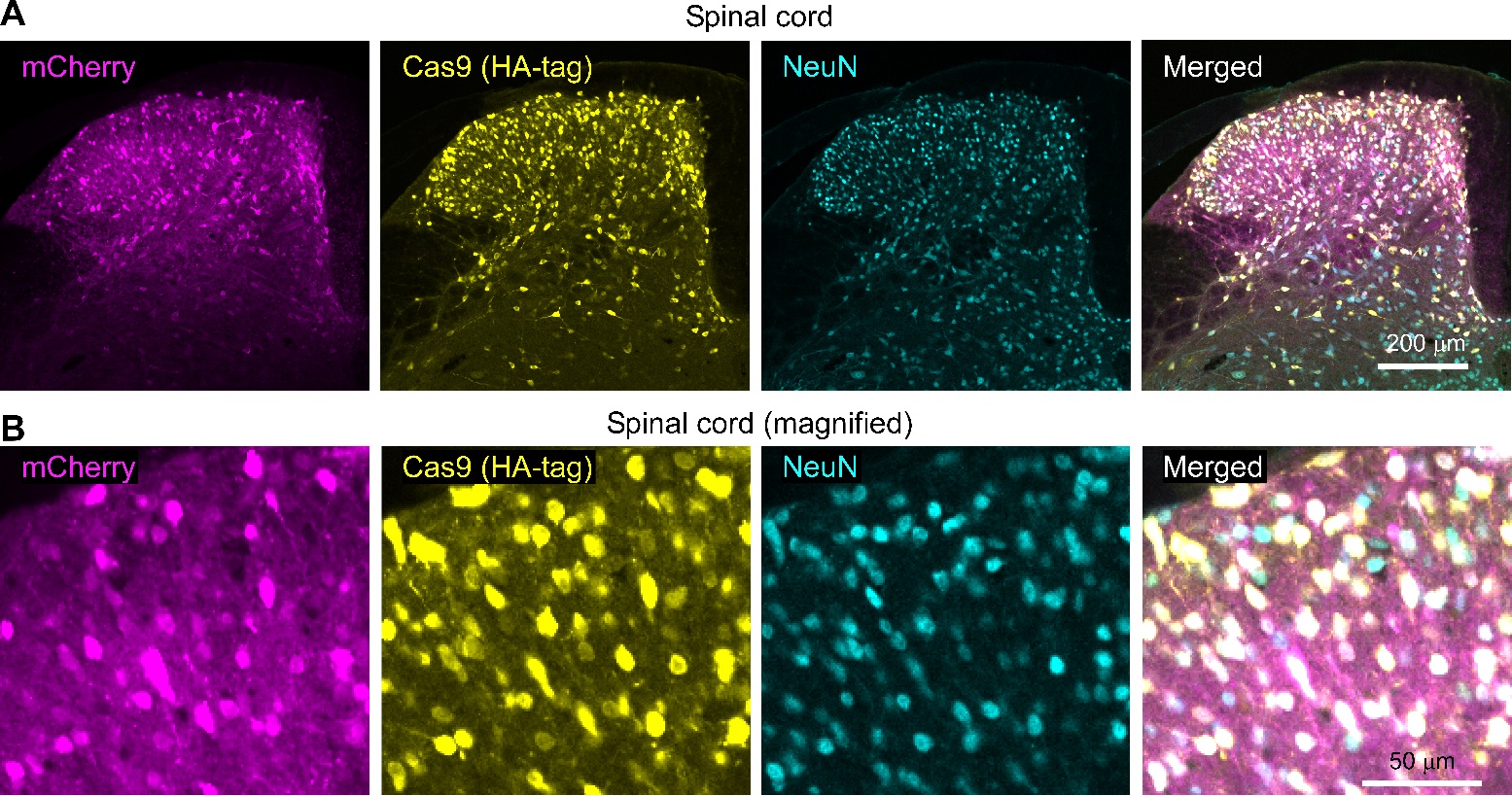
**

**Supplemental Figure 1. mCherry and Cas9 co-localization in spinal dorsal horn of sgCacna2d1 mice.** (A) Representative mages showing mCherry and Cas9 expression in spinal dorsal horn (mCherry, magenta; Cas9 (HA-tag), yellow; NeuN, cyan). (B) Magnified images of (A)


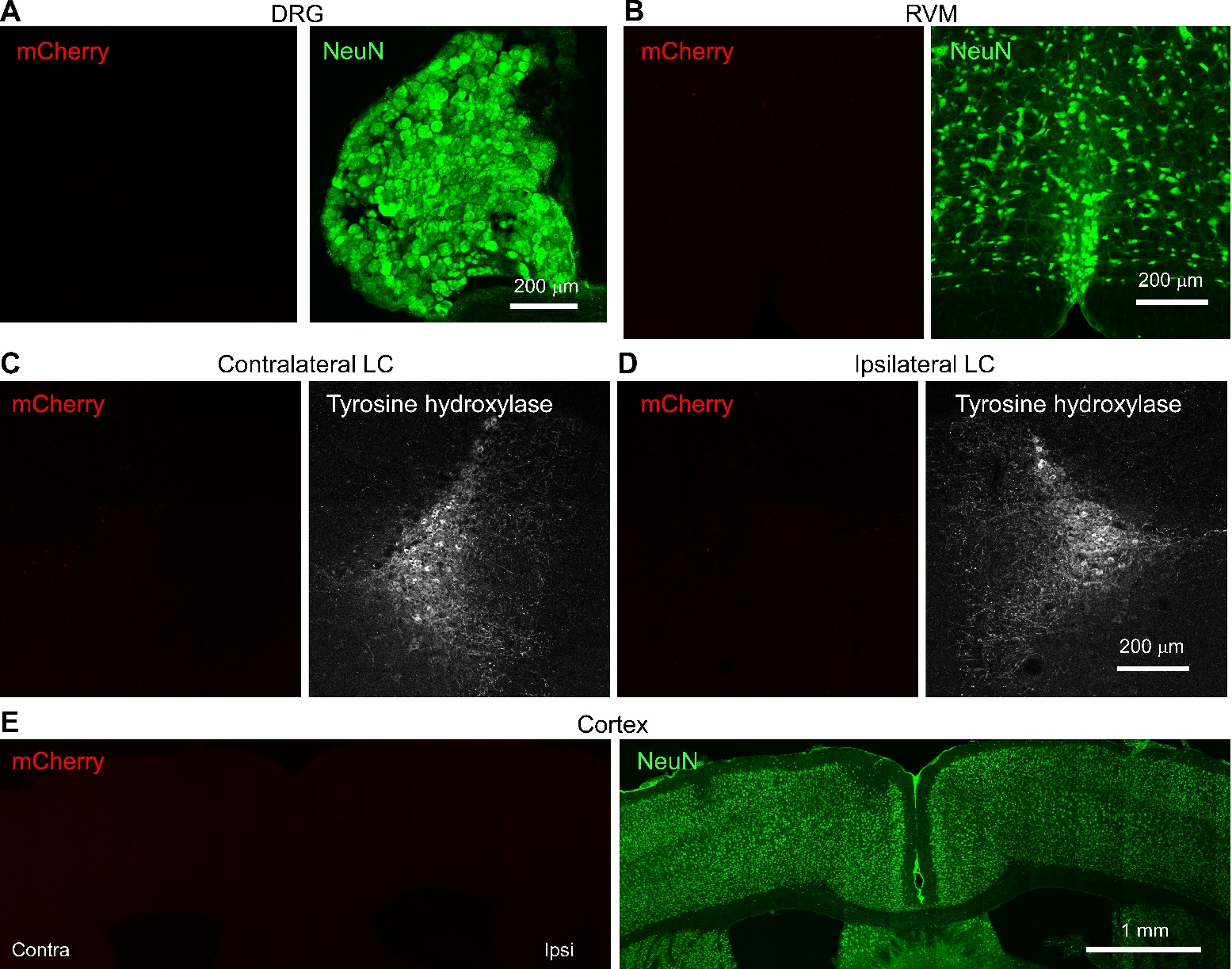


**Supplemental Figure 2. mCherry expression in dorsal root ganglion (DRG) and the brain regions sending input to spinal cord in sgCacna2d1 mice.** (A) mCherry expression in the L4 DRG ipsilateral to microinjection (mCherry, red, *left*; NeuN, green, *right*). (B) mCherry expression in the rostral ventromedial medula (RVM) (mCherry, red, *left*; NeuN, green, *right*). (C, D) mCherry expression in the loucus coeuleus (LC) contralateral (C) and ipsilateral (D) to the spinal cord injection (mCherry, red, *left*; tyrosine hydroxylase, grey, *right*). (A) mCherry expression in cortical regions including the somatosensory cortex and the morter cortex (mCherry, red, *left*; NeuN, green, *right*).


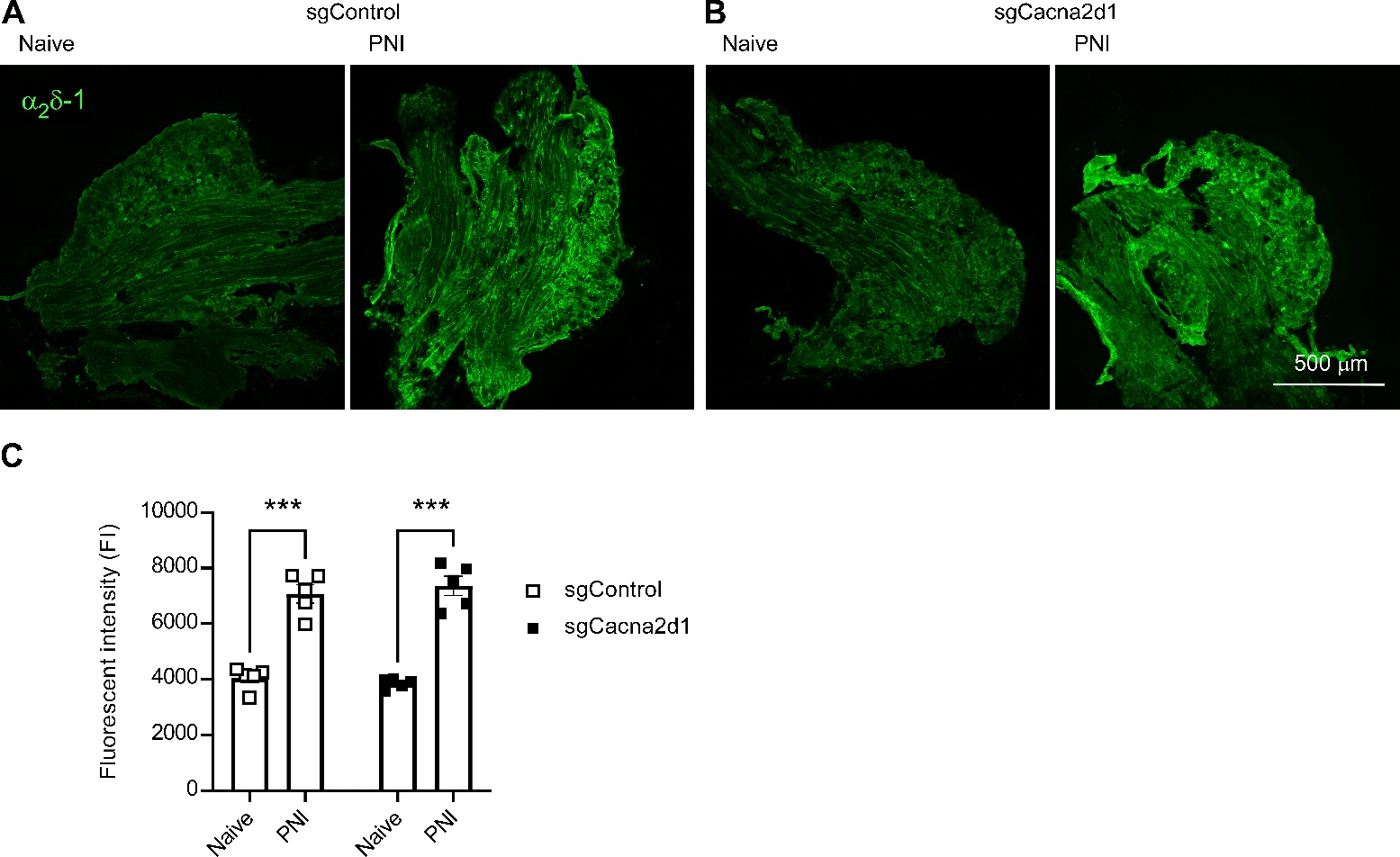


**Supplemental Figure 3. α_2_δ-1 upregulation in dorsal root ganglion (DRG) after PNI in sgControl and sgCacna2d1 mice.** (A) Images showing α_2_δ-1 expression in L4 DRGs of naïve (*left*) and PNI (*right*) sgControl mice (α_2_δ-1, green). (B) Same as (A) but those of naïve (*left*) and PNI (*right*) sgCacna2d1 mice. (C) Summary of α_2_δ-1 expression after PNI. (n = 5 each, two-way ANOVA post-hoc Bonferroni’s test, Group, F_(1, 16)_ = 0.0251, *p* = 0.876, Treatment, F_(1, 16)_ = 159.3, *p <* 0.0001, Interaction F_(1, 16)_ = 0.866, *p* = 0.366, ****p* < 0.001). Data are mean ± SEM.


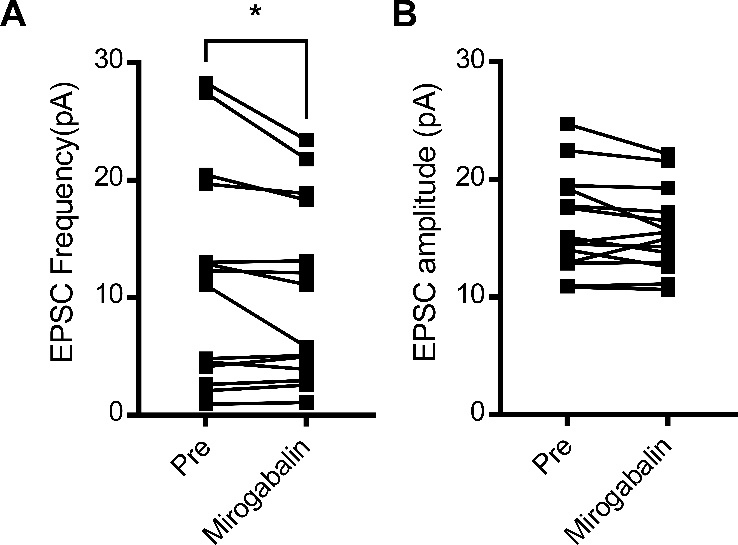


**Supplemental Figure 4. The mEPSC frequency after PNI were reduced by mirogabalin application.** (A, B) Summary showing the mEPSC frequency (A: n = 14 each , two-tailed paired *t-*test, t = 2.24, df = 13, **p < 0.05*) and the mEPSC amplitude (C: n = 14 each, two-tailed paired *t-*test, t = 1.71, df = 13, p = 0.112).


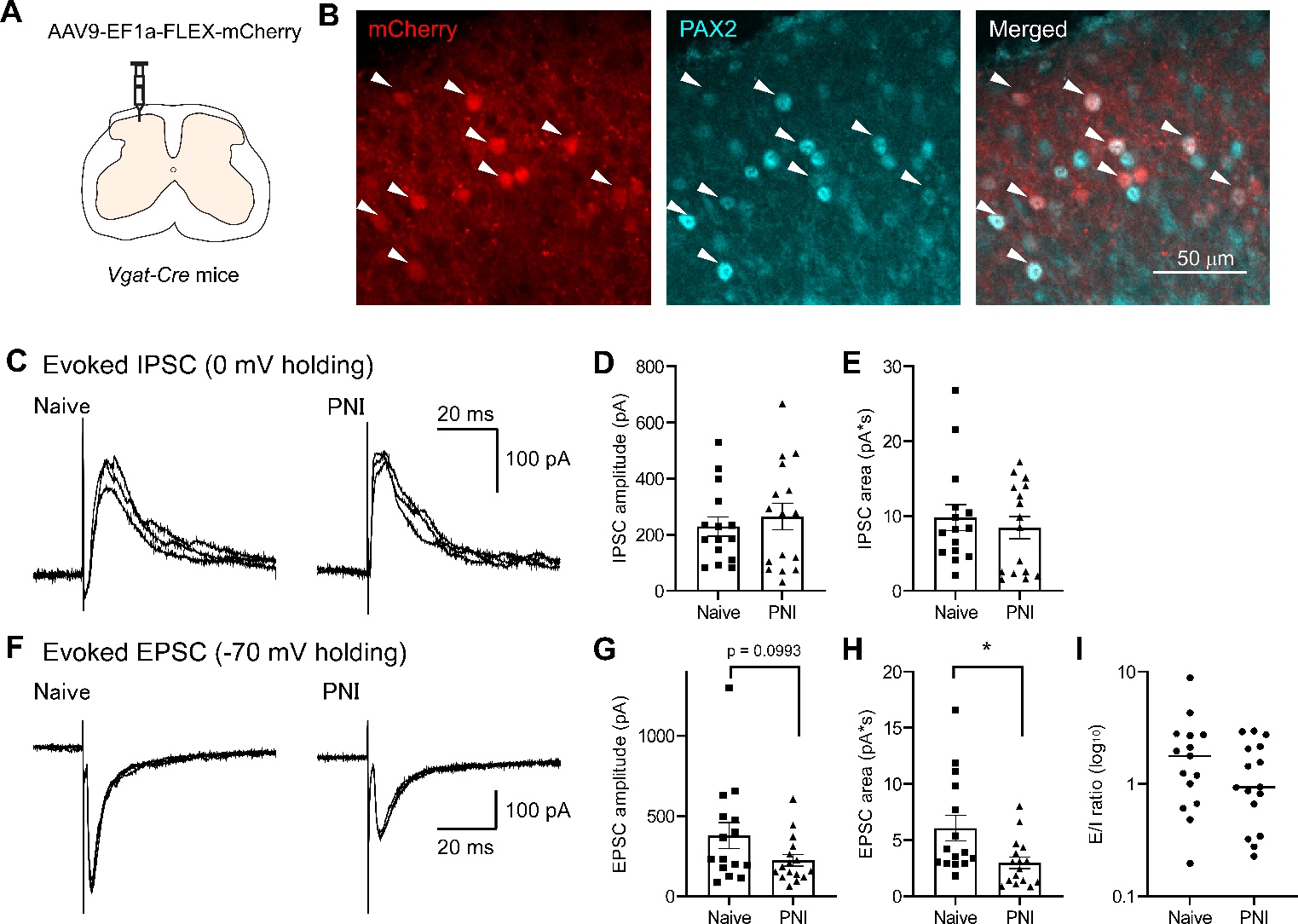


**Supplemental Figure 5. The evoked EPSCs in SDH inhibitory neurons were decreased after PNI.** (A) Schematic of the strategy to fluorescently label SDH inhibitory neurons with Cre-dependent AAV virus (AAV9-EF1α-FLEX-mCherry) injection and inhibitory neuron-specific Cre-expressing *Vgat-Cre* mice. (B) Representative images showing mCherry expression in the inhibitory neuron-labeled mice (mCherry, red; PAX2, a spinal inhibitory neuronal marker, cyan). (C) Representative traces of evoked IPSCs in SDH inhibitory neurons of naïve (*left*) or PNI (day 6–10, *right*) mice. (D, E) Summary of the IPSC amplitude of naïve and PNI mice (D; n = 15 (naïve), n = 16 (PNI), unpaired *t*-test, *t* = 0.596, df = 29, *p* = 0.556) and the IPSC area (E; n = 15 (naïve), n = 16 (PNI), unpaired *t*-test, *t* = 0.592, df = 29, *p* = 0.559). (F–J) Same as (C–E) but those of the evoked EPSCs in the inhibitory neurons. Representative traces (F), Summary of the EPSC amplitude (G; n = 15 (naïve), n = 16 (PNI), unpaired *t*-test with Welch’s collection, *t* = 1.73, df = 19.4, *p* = 0.099) and Summary of the EPSC area (G; n = 15 (naïve), n = 16 (PNI), unpaired *t*-test with Welch’s collection, *t* = 2.48, df = 19.9, **p < 0.05*). (H) Summary of the E/I ratio of naïve and PNI mice (n = 15 (naïve), n = 16 (PNI), unpaired *t*-test with Welch’s collection, *t* = 1.40, df = 19.2, *p* = 0.177). Data are mean ± SEM.


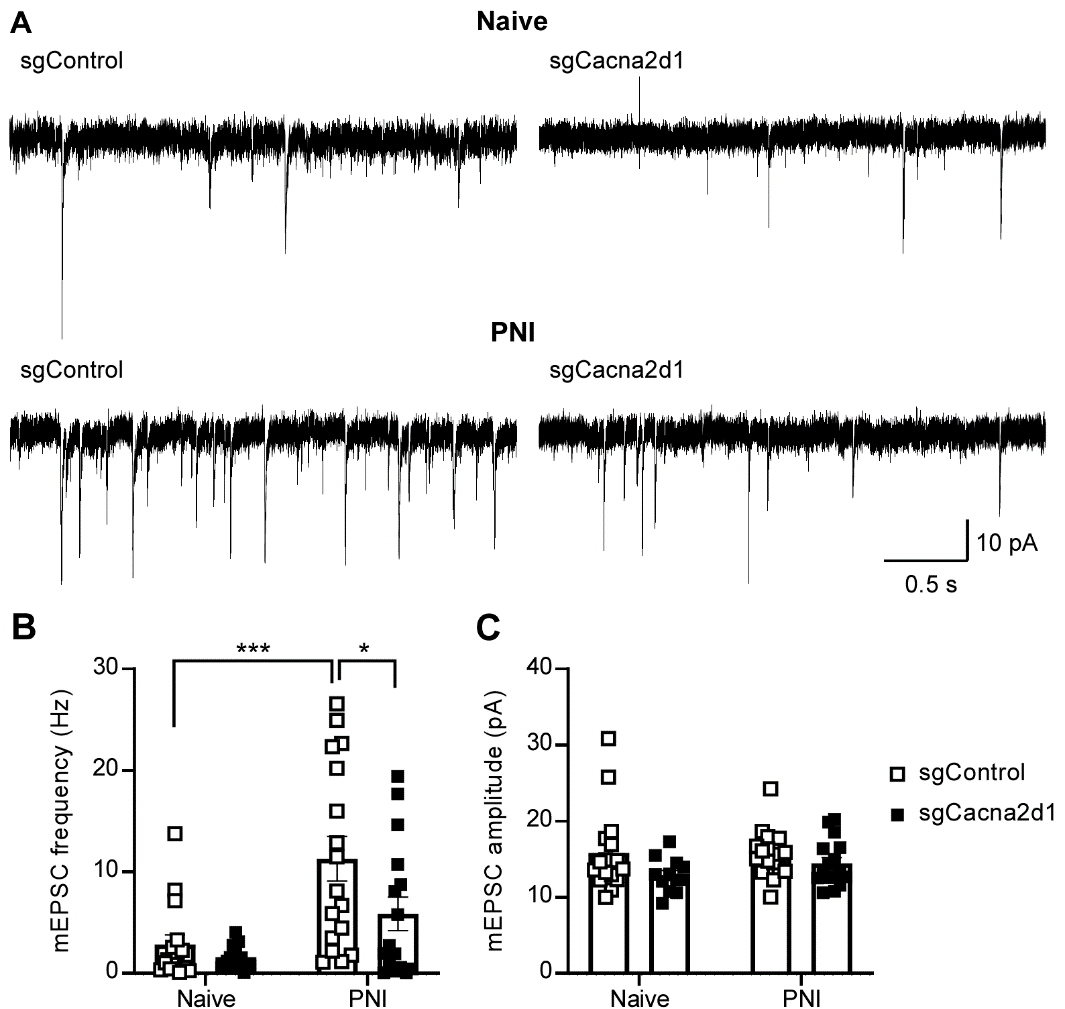


**Supplemental Figure 6.** **The facilitated mEPSC frequency after PNI were ameliorated in sgCacna2d1 mice.** (A) Representative traces showing mEPSCs in SDH neurons of sgControl (*left traces*) or sgCacna2d1 mice (*right traces*) without (Naïve, *upper traces*) or with PNI (*lower traces*). (B, C) Summary showing the mEPSC frequency (B: n = 16 (naïve, sgControl), n = 12 (naïve, sgCacna2d1), n= 17 (PNI, sgControl), n = 16 (PNI, sgCacna2d1), two-way ANOVA post-hoc Bonferroni’s test, Group, F_(1, 57)_ = 4.32, *p* = 0.0422, Treatment, F_(1, 58)_ = 15.6, *p* = 0.0002, Interaction F_(1, 57)_ = 1.68, *p* = 0.200, **p* < 0.05, ****p* < 0.001) and the mEPSC amplitude (C: n = 16 (naïve, sgControl), n = 12 (naïve, sgCacna2d1), n= 17 (PNI, sgControl), n = 16 (PNI, sgCacna2d1), two-way ANOVA post-hoc Bonferroni’s test, Group, F_(1, 57)_ = 4.69, *p* = 0.0345, Treatment, F_(1, 58)_ = 0.527, *p* = 0.471, Interaction F_(1, 57)_ = 0.800, *p* = 0.375).


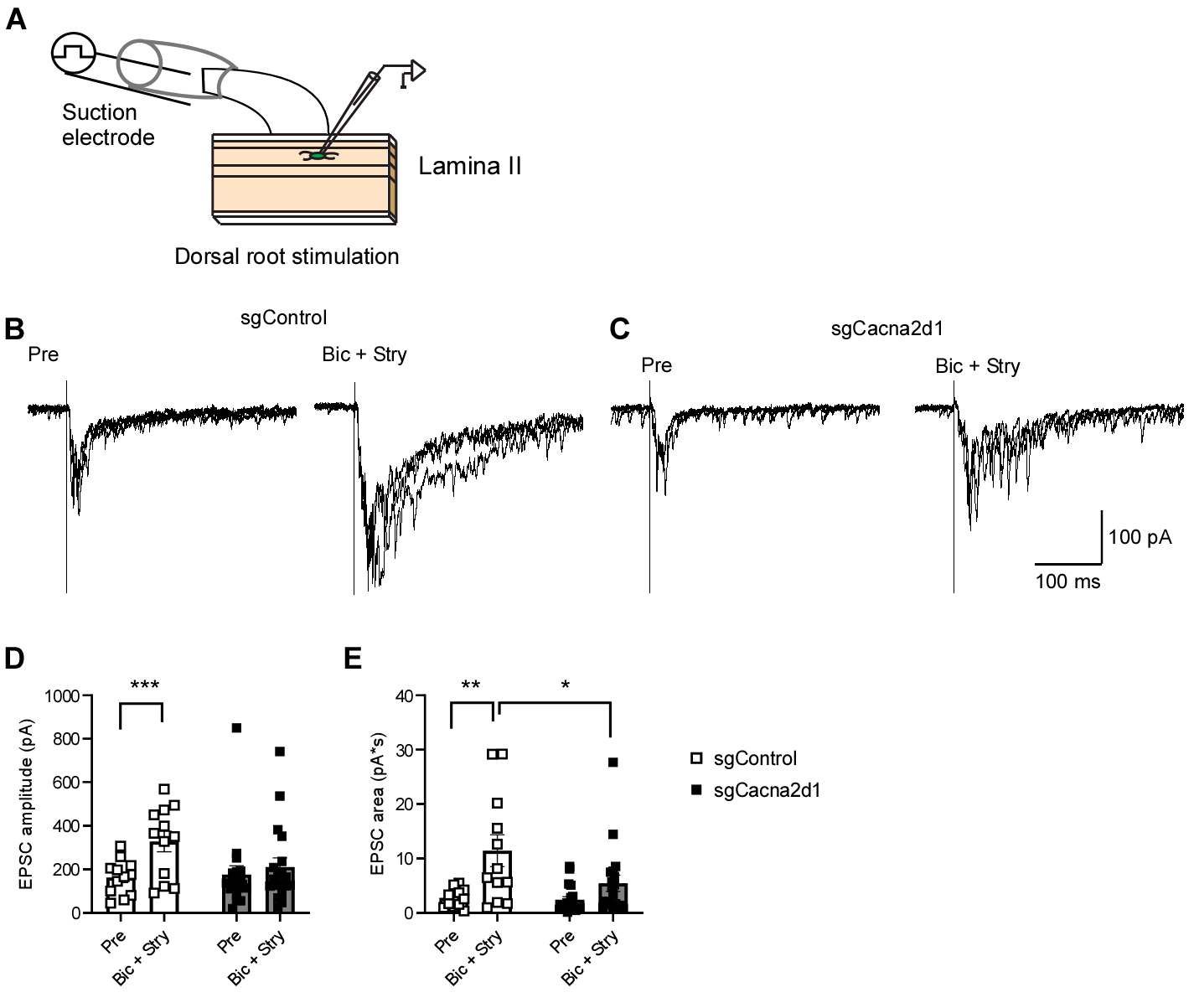


**Supplemental Figure 7. A fiber-induced EPSC facilitation by disinhibition were attenuated in sgCacna2d1 mice.** (A) Schematic of the whole-cell patch-clamp recording from SDH neurons and dorsal root stimulation with a suction electrode. (B) Representative traces showing A fiber-evoked EPSCs in SDH neurons of sgControl (B) or sgCacna2d1 mice (C) before (*left*) and after bicuculine (20 μM) and strychnine (2 μM) application (Bic + Stry, *right*). (D, E) Summary showing eEPSC amplitude of each group (D: n = 12 (sgControl), n = 19 (sgCacna2d1), two-way repeated measurements ANOVA post-hoc Bonferroni’s test, Group, F_(1, 29)_ = 0.905, *p* = 0.349, Treatment, F_(1, 29)_ = 18.6, *p* = 0.0002, Interaction F_(1, 17)_ = 7.93, *p* = 0.0087, ****p* < 0.001) and eEPSC area of each group (E: n = 12 (sgControl), n = 19 (sgCacna2d1), two-way repeated measurements ANOVA post-hoc Bonferroni’s test, Group, F_(1, 29)_ = 3.71, *p* = 0.064, Treatment, F_(1, 29)_ = 17.0, *p* = 0.0003, Interaction F_(1, 17)_ = 3.92, *p* = 0.0574, **p* < 0.05, ***p* < 0.01). Data are mean ± SEM.
